# Toxic Kidney Damage in Rats Following Subchronic Intraperitoneal Exposure to Element Oxide Nanoparticles

**DOI:** 10.3390/toxics11090791

**Published:** 2023-09-19

**Authors:** Yuliya V. Ryabova, Ilzira A. Minigalieva, Marina P. Sutunkova, Svetlana V. Klinova, Alexandra K. Tsaplina, Irene E. Valamina, Ekaterina M. Petrunina, Aristides M. Tsatsakis, Charalampos Mamoulakis, Kostas Stylianou, Sergey V. Kuzmin, Larisa I. Privalova, Boris A. Katsnelson

**Affiliations:** 1 Yekaterinburg Medical Research Center for Prophylaxis and Health Protection in Industrial Workers, 620014 Yekaterinburg, Russia; 2 Department of Pathology, Ural State Medical University, 620028 Yekaterinburg, Russia; 3 Department of Forensic Sciences and Toxicology, Faculty of Medicine, University of Crete, 71003 Heraklion, Greece; 4 Department of Human Ecology and Environmental Hygiene, IM Sechenov First Moscow State Medical University, 119991 Moscow, Russia; 5 Department of Urology, University General Hospital of Heraklion, Medical School, University of Crete, 71003 Heraklion, Greece; 6 Department of Nephrology, University General Hospital of Heraklion, Medical School, University of Crete, 71003 Heraklion, Greece; 7 Federal Budgetary Establishment of Science “F.F. Erisman Scientific Centre of Hygiene” of the Federal Service for Surveillance on Consumer Rights Protection and Human Wellbeing, 141014 Mytishchi, Russia

**Keywords:** kidney diseases, nanoparticles, nephrotoxicity, occupational exposure, oxides, urogenital system

## Abstract

Chronic diseases of the urogenital tract, such as bladder cancer, prostate cancer, reproductive disorders, and nephropathies, can develop under the effects of chemical hazards in the working environment. In this respect, nanosized particles generated as by-products in many industrial processes seem to be particularly dangerous to organs such as the testes and the kidneys. Nephrotoxicity of element oxide particles has been studied in animal experiments with repeated intraperitoneal injections of Al_2_O_3_, TiO_2_, SiO_2_, PbO, CdO, CuO, and SeO nanoparticles (NPs) in total doses ranging from 4.5 to 45 mg/kg body weight of rats. NPs were synthesized by laser ablation. After cessation of exposure, we measured kidney weight and analyzed selected biochemical parameters in blood and urine, characterizing the state of the excretory system. We also examined histological sections of kidneys and estimated proportions of different cells in imprint smears of this organ. All element oxide NPs under investigation demonstrated a nephrotoxic effect following subchronic exposure. Following the exposure to SeO and SiO_2_ NPs, we observed a decrease in serum creatinine and urea, respectively. Exposure to Al_2_O_3_ NPs caused an increase in urinary creatinine and urea, while changes in total protein were controversial, as it increased under the effect of Al_2_O_3_ NPs and was reduced after exposure to CuO NPs. Histomorphological changes in kidneys are associated with desquamation of the epithelium (following the exposure to all NPs except those of Al_2_O_3_ and SiO_2_) and loss of the brush border (following the exposure to all NPs, except those of Al_2_O_3_, TiO_2_, and SiO_2_). The cytomorphological evaluation showed greater destruction of proximal sections of renal tubules. Compared to the controls, we observed statistically significant alterations in 42.1% (8 of 19) of parameters following the exposure to PbO, CuO, and SeO NPs in 21.1% (4 of 19)—following that, to CdO and Al_2_O_3_ NPs—and in 15.8% (3 of 19) and 10.5% (2 of 19) of indicators, following the exposure to TiO_2_ and SiO_2_ nanoparticles, respectively. Histomorphological changes in kidneys are associated with desquamation of epithelium and loss of the brush border. The cytomorphological evaluation showed greater destruction of proximal sections of renal tubules. The severity of cyto- and histological structural changes in kidneys depends on the chemical nature of NPs. These alterations are not always consistent with biochemical ones, thus impeding early clinical diagnosis of renal damage. Unambiguous ranking of the NPs examined by the degree of their nephrotoxicity is difficult. Additional studies are necessary to establish key indicators of the nephrotoxic effect, which can facilitate early diagnosis of occupational and nonoccupational nephropathies.

## 1. Introduction

Kidney disease is widespread throughout the world. Nephropathies often have a long asymptomatic latency period because kidneys have enormous compensatory capabilities and can maintain homeostasis for years. Chronic kidney disease has been estimated to affect 9% to 15% of the population in different regions of the world [1]. It has a major effect on global health, both as a direct cause of global morbidity and mortality and as an important risk factor for cardiovascular disease, but it is largely preventable and treatable and deserves greater attention in global health-policy decision making, particularly in locations with low and middle sociodemographic indexes [2,3]. Chronic diseases of the urogenital tract, including bladder cancer, prostate cancer, reproductive disorders, and nephropathies, can be induced by exposure to hazardous chemicals at work [4,5,6,7,8,9,10,11,12]. Kidneys are particularly susceptible to the adverse effects of chemical pollutants. This organ filters almost 200 L of blood per day, producing up to 2 L of urine. As a result, pollutants have a strong impact on kidneys [13]. In their recent review, Lentini et al. [14] revealed correlations between acute and chronic kidney disease and environmental levels of heavy metals and other risk factors. The main components of the aerosol polluting the workplace air in the production of aluminum titanium alloys are Al, Ti, and Si [15]. Occupational exposure to selenium, copper, and their compounds occurs in metallurgy during copper sludge processing; roasting of copper pyrites; and manganese, selenium, and tellurium production [16,17]. The sources of environmental pollution with lead and cadmium, in addition to the mining and metallurgical industries, are the production of batteries and electroplating, urban road dust, cigarette smoke, etc. [18,19]. Besides this, workplace air pollution with metal oxide submicron and nanosized compounds generated as by-products is common for many industries. Understanding the mechanisms of nephrotoxicity of nanoparticles (NPs) can serve as a tool for the early diagnosis of occupational nephropathies, enabling timely treatment and a longer work ability of humans. The current scientific literature presents experimental studies on the damaging effect of NPs on the structure and function of the testes and kidneys following exposure through different routes [20,21,22,23,24,25,26,27,28,29].

The general and specific toxic effects of Al_2_O_3_, TiO_2_, SiO_2_, PbO, and CdO NPs, as well as cytotoxic effects of CuO and SeO NPs, were discussed in detail in our previous works [15,30,31,32,33]. We have noted that Al_2_O_3_, TiO_2_, SiO_2_, PbO, and CdO NPs exhibit multiple organ toxicity, including certain signs of kidney damage, on which we want to focus in this paper. The aim of the present study was to analyze nephrotoxic effects of subchronic exposures to NPs based on weight, biochemical, histomorphometric, and cytological indicators.

## 2. Materials and Methods

Suspensions of the element oxide (EO) nanoparticles (NPs) were prepared at the Center for Collective Use “Modern Nanotechnologies” of the Ural Federal University, Yekaterinburg, using laser ablation of thin-sheet targets of the corresponding material of 99.99% purity in sterile deionized water. The technique has been described in more detail elsewhere [13,14]. The stability of suspensions was characterized by their zeta potential, measured using the Zetasizer Nano ZS analyzer (Malvern Panalytical Ltd., Malvern, UK), and was found to be high (up to 42 mV), thus enabling an increase in particle concentrations up to 0.5 g/L by partial evaporation of water at 50 °C, without changing the size and chemical characteristics of EO NPs. The particle shape and size were characterized using scanning electron microscopy (SEM) and particle size distribution curves (d) (Table 1).

Experimental studies were conducted on outbred male albino rats aged 3 to 4 months at the beginning of the experiment. The weight variations of animals did not exceed ±20% of the mean weight of all the animals at the commencement of the studies. Repeated intraperitoneal injections thrice per week for six weeks (18 injections in total) were used to simulate subchronic toxicity. The animals were kept, fed, cared for, and removed from the experiment in strict accordance with the International Guiding Principles for Biomedical Research Involving Animals developed by CIOMS and ICLAS (2012). The animal study protocols were approved by the Institutional Ethics Committee of the Yekaterinburg Medical Research Center for Prophylaxis and Health Protection in Industrial Workers (Table 2). The choice of single doses of NPs under study was limited by the maximum concentration of stable NP suspensions and by the individual tolerance of animals established in previous pilot experiments. The exposure doses selected for the experiments induced moderate toxicity without causing pain to or the death of the animals.

Before euthanasia, 24-h urine specimens were collected to measure the urinary pH, protein, levels of urea, uric acid, and creatinine and to calculate endogenous creatinine clearance.

After exposure cessation, blood samples were taken by cervical dislocation during euthanasia and then tested for creatine kinase, uric acid, urea, and creatinine in blood serum, using Cobas Integra 400 plus automated analyzer (Roche Diagnostics GmbH, Germany) with appropriate test kits: CK Creatine Kinase COBAS INTEGRA/cobas c system, UA2 Uric Acid ver.2 COBAS INTEGRA/cobas c system, UREAL Urea BUN COBAS INTEGRA/cobas c system, and CREJ2 Creatinine Jaffe Gen. 2 COBAS INTEGRA/cobas c system (Roche Diagnostics GmbH).

The animals were dissected immediately after euthanasia, and their kidneys were visually examined and weighed. Imprint smears were made from fresh kidney sections and stained with Leishman for air-dried smears. The cellular composition and signs of cell damage were assessed using a Carl Zeiss Primo Star binocular microscope with a USCMOS video camera imaging system at 100× and 1000× magnification. During the microscopy, 300 cells from the imprint were counted.

Histological specimens were prepared by immersing kidneys in formalin and then cutting them into 2–3 mm thick slices treated with alcohols of increasing concentration and embedding in paraffin. Then, 3–4 μm sections were cut from the embedded blocks and stained with hematoxylin and eosin; in addition, the method of periodic acid–Schiff stain was also used. The study of histological preparations and their microphotography and morphometry were carried out using the Avtandilov ocular measuring grid and a computer software for pattern recognition, using an Olympus CX-41 microscope with an Olympus Soft Imaging Solution GMBH, Model LC20 camera, and LCmicro software. At least 30 measurements of each indicator were taken on preparations of four rodents from each exposure and the control groups.

Statistical analyses were performed using SPSS software (IBM Corp. Released 2021. IBM SPSS Statistics for Windows, Version 28.0. IBM Corp., Armonk, NY, USA). Groups were compared using one-way analysis of variance (ANOVA), followed by a normality check with the Shapiro–Wilk test. Post hoc comparisons were performed using the Bonferroni test. A *p*-value ≤ 0.05 was considered significant.

## 3. Results

In the absence of significant changes in the body weight (BW) of exposed animals compared to controls, the kidney weight showed a statistically significant decrease in those administered Al_2_O_3_ NPs (by 8% in proportion to BW); it showed an increase in those exposed to PbO NPs (by 9% of the absolute weight); and it remained unchanged following the exposure to SeO, CuO, TiO_2_, SiO_2_, and CdO NPs in the corresponding experiments.

The administration of SiO_2_ and SeO NPs significantly reduced concentrations of urea and creatinine in blood serum, respectively (Table 3 and Appendix A Table A1).

No statistically significant changes were observed in the 24-h urine volumes and pH values (Table 1) in any group. However, we observed a statistically significant decrease in urinary total protein under the effect of CuO NPs and an increase in creatinine and urea concentrations after subchronic exposure to Al_2_O_3_ NPs (Table 3 and Appendix A Table A1). The comparative analysis of biochemical test results showed that Al_2_O_3_ NPs had the most pronounced nephrotoxic effect.

**Figure 1:**
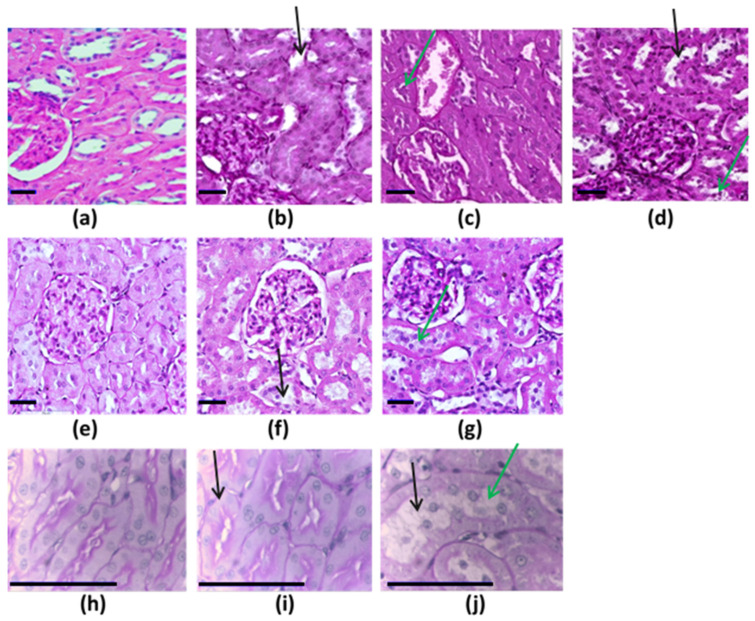
Histological picture of kidneys in control and EO-NP-exposed animals, periodic acid–Schiff staining, a 90× magnification for top and middle rows, and 400× magnification for bottom row. (**a**) Controls in the experiment with Al_2_O_3_, TiO_2_ and SiO_2_ NPs; animals exposed to (**b**) Al_2_O_3_ NPs, (**c**) TiO_2_ NPs, and (**d**) SiO_2_; (**e**) controls in the experiment with CdO and PbO NPs; animals exposed to (**f**) CdO NPs and (**g**) PbO NPs; (**h**) controls in the experiment with SeO and CuO NPs; and animals exposed to (**i**) SeO NPs and (**j**) CuO NPs. Black scale bars—35 mkm. The black arrow points at dilated renal tubules, and the green arrow marks pronounced dystrophic and necrobiotic changes.

The morphological picture of kidneys in control animals from all experimental studies corresponded to the histological norm: the epithelium of convoluted tubules of the kidney had a clear periodic acid–Schiff (PAS) positive brush border on the apical side, homogenous cytoplasm, and optically clear nuclei (Figure 1). At the same time, we observed such dystrophic changes of various severity as dilated tubular lumens and deformed glomeruli in the exposed animals.

**Figure 2:**
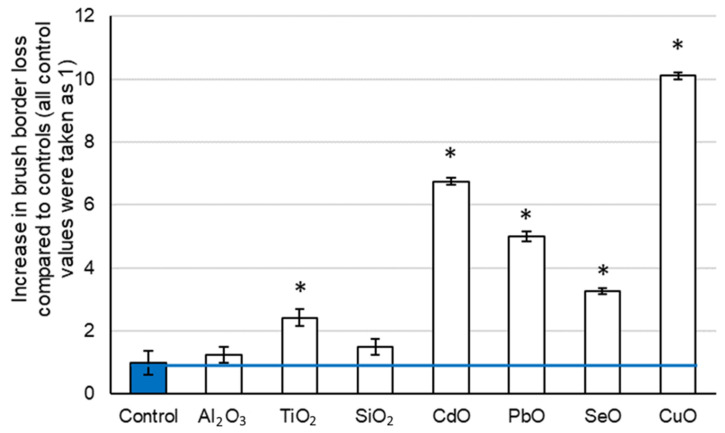
Loss of the brush border in rat kidney tissues following exposure to selected element oxide nanoparticles compared to the controls; the value of the indicator in each control group was taken as 1; values of the indicator in other groups were normalized to the control. Statistically significant difference from the corresponding values in *—control group (*p* < 0.05 via Student’s *t*-test).

**Figure 3:**
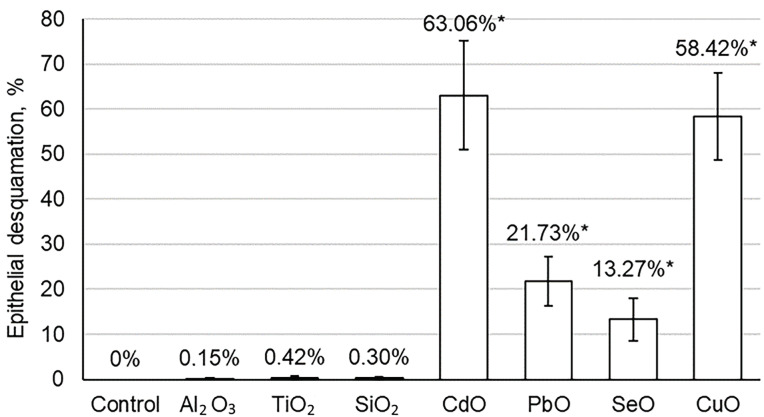
Percentage of desquamated epithelial cells in the kidneys of the rats exposed to selected element oxide nanoparticles compared to controls. Statistically significant difference from the corresponding values in *—control group (*p* < 0.05 via Student’s *t*-test).

The histomorphological evaluation of the kidney tissues of animals exposed to EO NPs showed dystrophic changes of varying severity in the renal tubular epithelium: from the destruction of the brush border of epithelial cells to areas of necrobiosis/desquamation on the epithelium. Differences in morphometric parameters with the control animals were statistically significant in almost all groups (Figure 2 and Figure 3). Cytological lesions were observed in all experimental groups following exposure to NPs (Table 4) and assessed in kidney imprint smears. A significantly larger proportion of lesions was found in proximal, rather than in distal, parts of the tubules.

## 4. Discussion

The current scientific literature presents several experimental studies on the damaging effect of NPs on the structure and function of the testes and kidneys following exposure through different routes [20,21,22,23,24,25,26,27,28,29,30,34]. Male rodents’ in vitro and in vivo reproductive toxicity caused by different types of inorganic NPs (AgNPs, AuNPs, IONPs, ZnONPs, TiO_2_NPs and NiNPs) was recently evaluated in a systematic review of data published in the last twelve years. Structural and functional alterations were commonly observed in Sertoli, Leydig, germ, and sperm cells in vitro and in vivo. Oxidative stress, apoptosis, and/or necrosis were the most common findings after inorganic NP exposure. The toxicity of different NPs depends strongly on their physicochemical characteristics/intrinsic properties [34]. An oral route for 20 days of NPs of nano-silicon dioxide (nano-SiO_2_) at a dose of 600 and 900 mg/kg/day was found to induce hyperemia in the kidney [21]. The study of a silver selenide nanocomposite at a dose of 500 μg/kg revealed swelling of renal convoluted tubules and, as a consequence, the compression of renal glomeruli and the proliferation of connective tissue in the interstitial space of renal tubules, a typical sign of kidney sclerosis [22]. Histological alterations in kidneys have been shown following a 28-day oral administration of iron oxide NPs. A higher concentration (1000 μg/kg) induced the dystrophy of convoluted tubules and a plethora of vessels of the renal cortex and glomeruli [23]. A morphological study of kidneys in rats orally exposed to Au NPs showed no significant differences between the animals administered NPs of different sizes: all of them developed dystrophy of renal tubular epithelium and focal cell necrosis. In addition, such changes in the lumen of convoluted tubules as a narrowing, stellate appearance and desquamated epithelium (in some fields of view) were observed, along with a moderate expansion of capillary loops of the glomeruli and plasma impregnation in small arterioles [24]. Other authors demonstrated the development of hyperemia of the renal tubules in Wistar rats after a 7-day oral administration of chromium oxide NPs at a dose of 500 μg/kg, as well as the cellularity of glomerular capillaries on day 14. The dose of 2000 μg/kg administered during the 7 days caused tubular hyperemia, as well as focal tubular atrophy and necrosis of the tubular epithelium. The histological picture observed following 14 days of a high-dose exposure was characterized by alterations in the kidney architecture, as evidenced by thickened capillary walls and the obliteration of the Bowman’s capsule [25]. Inhalation exposure of mice to lead NPs at a concentration of 0.956 × 10^6^ NPs/cm^3^, yielding a total dose of 1.684 μg/g body weight, over 11 weeks showed the highest accumulation of lead in the kidneys, while their histological examination revealed large lipid vacuoles in some blood vessels. The renal tubules that were closely adjacent to such vessels were compressed and showed damage to the epithelial membrane. Accumulations of lead NPs were found in the epithelial cells of proximal renal tubules [26]. The 7-day oral exposure to ultrafine lead oxide NPs at doses of 5 mg/kg and 10 mg/kg induced the loss of renal cell architecture and the narrowing of the inner diameter of both proximal and distal convoluted tubules [27].

Thus, in most studies, exposure to inorganic nanoparticles, regardless of the route of administration, led to structural and functional kidney disorders, among which oxidative stress, apoptosis and/or necrosis, hyperemia, and the destruction of the epithelium were most often described.

Kidneys are the main organ for the elimination of toxicants from the bloodstream [13]. NPs are known to increase oxidative stress by promoting the formation of reactive oxygen species [35]. Alterations in oxidative stress markers in the blood of animals (increased MDA and decreased catalase activity) were observed to a certain extent after exposure to all NPs examined [15,30,31,32,33]. Our studies aimed at showing the universal danger of NPs. Not all of the chemicals tested exert a toxic effect in their soluble form. Selenium, for instance, is an essential element in the physiological range of doses [33]. Titanium compounds have no adverse renal effects [36]. At the same time, inorganic compounds of aluminum [37], silicon [38], lead [39], cadmium [40,41], and copper [42] are nephrotoxic. However, they are all toxic in the form of NPs. In our research, all NPs were found to have a certain nephrotoxic effect. Biochemical, cytomorphological, and histological parameters did not always change in an interconnected manner, a fact that somewhat complicates the comparative assessment of the nephrotoxic effects of the nanosized particles under study.

According to the number of parameters of rats’ urine and serum (urine pH shift to the acidic side and an increase in the content of protein, urea, uric acid, and creatinine in it at reduced mass coefficients of both kidneys), the Al_2_O_3_ NPs’ effect was the most pronounced compared to that of other NPs administered, despite a relatively low dose of these NPs, which indirectly indicates their greater toxicity compared to TiO_2_, SiO_2_, CuO, and SeO NPs. Titanium dioxide NPs at doses of 300 mg/kg body weight (by gavage for 3 weeks) caused a 2- and 1.6-fold increase in concentrations of urea and serum creatinine, respectively [20]. We observed a similar, yet weaker, increase due to a significantly lower dose of NPs. We found a decrease in serum urea and creatinine after all NPs (excluding CdO and PbO NPs). It may indicate an increasing glomerular filtration rate. Previously, after exposure to SiO_2_ NPs, hyperemia was shown [21]. It may be associated with an increased glomerular filtration rate and a decrease in serum urea and creatinine, which we observed in our study. A chronic increase in the glomerular filtration rate is a known cause of glomerulosclerosis and eventually leads to the loss of kidney function. However, the loss of kidney function has not taken place in our study.

It is worth noting that an even lower dose of CdO NPs, as well as the highest dose of PbO NPs, caused no significant changes in urine and serum parameters in rats compared to other NPs. PbO NPs increased both the absolute and relative weight of kidneys. We assume that NPs of the elements less demanded by the body have led to lesser changes in the urine and blood of rats due to the absence of natural mechanisms for the entry of nonessential metal ions into the cell. The size of nanoparticles is known to affect their toxicity, but an inverse monotonic relationship between them has not been shown by all nanoparticles. Even the finest nanoparticles (<10 nm) do not always demonstrate the highest toxicity. In the range from 10 to 100 nm, toxicity also often changes nonmonotonically [43,44]. Easy cellular uptake of NPs related to their size has been demonstrated [45]. It is believed that nanoparticles ≥50 nm are more easily recognized by defense systems of the body, which attenuates their toxic effect [43]. In our study, however, no such trend was observed, because rather large CdO and PbO NPs demonstrated more pronounced nephrotoxicity than smaller Al_2_O_3_ and TiO_2_ NPs.

A statistically higher percentage of brush border loss in the rats exposed to TiO_2_ NPs compared to the control animals, in contrast to those exposed to Al_2_O_3_ and SiO_2_ NPs, was detected in the present study (Figure 2 and Appendix A Table A2). The same trend was noticeable in terms of epithelial desquamation (Figure 3 and Appendix A Table A2). If minimal changes in the histomorphometric parameters of the kidneys in rats exposed to Al_2_O_3_ NPs can be partly explained by their lower dose, then in this trinity, TiO_2_ NPs are the most nephrotoxic in terms of histomorphometric parameters.

Experimental animals exposed to PbO and CdO NPs demonstrated an even greater loss of the brush border. Cadmium and lead are known nephrotoxic poisons [40,41,46,47,48], and both of these metals are nonessential elements, so we expected to observe toxic damage to the kidneys after the cessation of exposure. The diameter of the renal glomeruli statistically decreased to the greatest extent (by 17.9%) in the rats exposed to PbO NPs (32.90 ± 1.01 µm against 40.08 ± 1.09 µm in the controls, *p* < 0.05), but tubular epithelium was more damaged in those exposed to CdO NPs, as seen by the loss of the brush border and desquamation of the epithelium both on histological slides and cytological changes on smear preparations (Figure 2 and Figure 3, Table 4, and Appendix A Table A2). The cell counts in imprint smears also indicated damage to the proximal rather than distal tubules.

In the experiment with SeO NPs and CuO NPs, a histological and cytomorphological assessment of their effects showed the greatest loss of the brush border induced by CuO NPs (Figure 2 and Appendix A Table A2). At the same time, the cytomorphological parameters showed a slightly greater deviation from the control values in the SeO NP exposure group (Table 4).

CuO NPs have a larger surface area and dissolve quickly, and dissolved copper easily changes its oxidation state [49]. All of these properties of CuO NPs lead to a sharp increase in oxidative stress in all organs of penetration, including the kidneys. NPs can be potentially excreted through kidneys, being transported outside the filtering glomerulus directly to the renal tubule [50,51].

Kidneys play an important role in selenium homeostasis [41,52]. Selenium is often used as a protector against nephrotoxic effects of chemicals (e.g., [53,54,55,56,57]). However, it has been shown that rising doses of Se NPs have an increasingly pronounced nephrotoxic effect and accumulate in renal tissues [58].

The results of cytology tests of kidney imprint smears (Table 4) were similar to those of the histological examination in terms of damages observed in all experimental groups (Figure 2 and Figure 3 and Appendix A Table A2). Damage is seen in the cells of the proximal (rather than distal) convoluted tubules, but at the same time, toxic inflammation of this organ is again indicated mainly by the eosinophilic reaction in cytological imprint smears.

It is most likely that these metals exert a toxic effect on kidneys not so much in the form of persistent EO NPs, but rather in the form of ions released in the course of particle dissolution in biological fluids. Therefore, it can be assumed that the particular nephrotoxicity of TiO_2_ NPs compared with Al_2_O_3_ and SiO_2_ NPs is explained by its highest solubility, which was shown when fetal bovine serum was added in vitro to each nanosuspension [15]. After the partial dissolution of NPs, their toxic effects are attributed to the damaging effect of the remaining undissolved NPs, on the one hand, and that of the dissolved ions of chemical elements on the other. In fact, a nanoparticle is, on the one hand, a damaging agent itself and, on the other, a long-term source of ions of a chemical element that makes up its core. Regarding the element oxide NPs tested, it is worth noting the nephrotoxicity of soluble forms of aluminum, silicon, and copper described elsewhere [59,60,61]. At the same time, selenium in a narrow dose range was used to attenuate As- or Cd-mediated toxicity in kidneys [62]. Titanium derivatives showed no signs of nephrotoxicity [15], were widely studied [63,64,65], and are now used in anticancer therapy [66]. Undissolved NPs are transported in the bloodstream to organs and tissues, thus reaching the kidneys. The relocation of nanoparticles from lungs to kidneys and liver has been observed following inhalation exposure to lead oxide nanoparticles in mice [67].

On the whole, we evaluated 19 parameters of the excretory system following the exposure to Al_2_O_3_, TiO_2_, SiO_2_, PbO, CdO, CuO, and SeO NPs. Compared to the controls, we observed statistically significant alterations in 42.1% (8 of 19) of parameters following the exposure to PbO, CuO, and SeO NPs, in 21.1% (4 of 19)—following that to CdO and Al_2_O_3_ NPs—and in 15.8% (3 of 19) and 10.5% (2 of 19) of indicators following the exposures to TiO_2_ and SiO_2_ NPs, respectively.

Although a direct comparison of nanoparticle nephrotoxicity in our study is impractical due to different doses under study, it is worth mentioning that PbO, CuO, and SeO NPs were found to have the most pronounced nephrotoxic effect, as judged by the alterations induced. It is of particular interest that the dose of CuO or SeO NPs was lower than that of PbO NPs, but their toxic effects were comparable.

Summarizing our findings, it is worth noting that, regardless of the chemical nature, element oxide NPs have a nephrotoxic effect and cause more damage to proximal (rather than distal) parts of renal tubules.

The common mechanisms of renal damage following the exposure of NPs of any chemical nature include an increase in oxidative stress, accumulations and penetrations of NPs into cells and damage to the intracellular structures, and toxic effects of chemical elements composing NPs after dissolution in biological fluids. Understanding the mechanisms of the renal toxicity of NPs can facilitate early diagnosis of occupational nephropathies, thus enabling timely treatment, slowing down the disease progression, and prolonging the human work capacity.

## 5. Conclusions

NPs generated as by-products in many industrial processes seem to be particularly dangerous to organs such as the testes and the kidneys. All element oxide NPs under investigation that were instilled intraperitoneally demonstrated a nephrotoxic effect, following subchronic exposure, in the present study. Unambiguous ranking of the NPs examined by the degree of their nephrotoxicity is difficult. The severity of cyto- and histological structural changes in kidneys depends on the chemical nature of NPs. These alterations are not always consistent with biochemical ones, thus impeding early clinical diagnosis of renal changes.

Additional profound research is necessary to establish molecular genetic aspects of exposure to nanoparticles of a different chemical nature, with a different accumulation and dissolution of nanoparticles in kidneys and other organs and different mechanisms of the cellular uptake of nanoparticles and their dissolved ions. It can also facilitate the identification of key indicators of nephrotoxic effects contributing to the early diagnosis of NP-induced occupational and nonoccupational nephropathies.

## Figures and Tables

**Table 1:** Description of the nanoparticles used in animal experiments.

EO NPs, Size, nm	SEM Image of EO NPs in Suspension (x)	Particle Size Distribution Curve
Al_2_O_3_ 21 ± 6	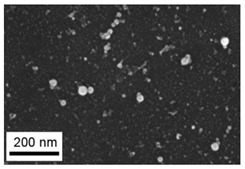	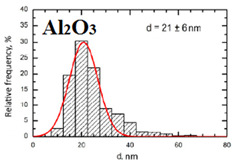
TiO_2_ 27 ± 7	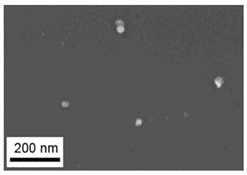	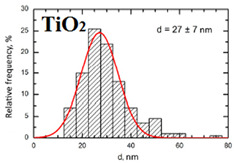
SiO_2_ 43 ± 11	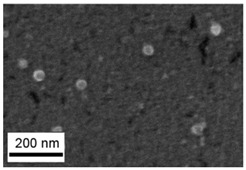	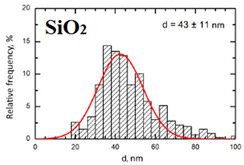
CdO 57 ± 13	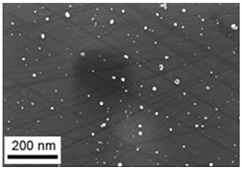	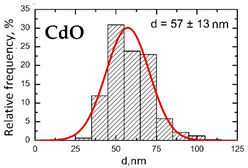
PbO 50 ±16	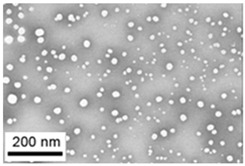	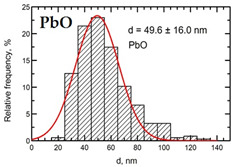
SeO 51 ± 14	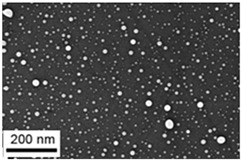	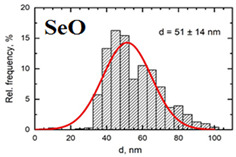
CuO 21 ± 4	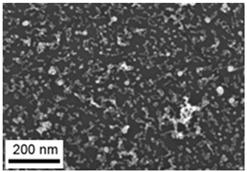	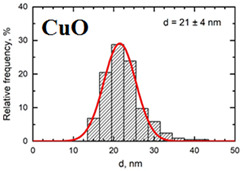

**Table 2:** Toxicity modeling in in vivo experiments.

AE	Group	Animals, *n*	Pre-Exposure Animal Body Weight, g	Injected Volume (mL), and Concentrations (mg/mL)	Total Dose of EO NPs Received	Local Ethics Committee Protocol No.
1	Control	12	292.27 ± 5.02	1 mL DIW	0 mg/kg	No. 62 of 1 February 2017
	Al_2_O_3_	12	289.55 ± 7.93	0.25 mg/mL	18 mg/kg	
	TiO_2_	12	287.50 ± 7.40	0.5 mg/mL	36 mg/kg	
	SiO_2_	12	285.91 ± 9.17	0.5 mg/mL	36 mg/kg	
2	Control	12	217.50 ± 7.57	2 mL DIW	0 mg/kg	No. 2 of 1 February 2018
	CdO	12	217.50 ± 6.36	0.2 mL of 0.25 mg/mL NP suspension (per 200 g BW, i.e., 0.25 mg/kg BW of NPs) + 1.8 mL DIW	4.5 mg/kg	
	PbO	12	222.75 ± 6.38	1 mL of 0.5 mg/mL NP suspension (per 200 g BW, i.e., 2.5 mg/kg BW of NPs) + 1 mL DIW	45 mg/kg	
3	Control	12	239.17 ± 4.12	4 mL DIW	0 mg/kg	No. 2 of 20 April 2021
	SeO	12	225.91 ± 3.62	2 mL of 0.25 mg/mL NP suspension + 2 mL DIW	36 mg/kg	
	CuO	12	230.67 ± 2.91	2 mL of 0.25 mg/mL NP suspension + 2 mL DIW	36 mg/kg	

Abbreviations: AE, animal experiment; DIW, deionized water; BW, body weight; NP, nanoparticles.

**Table 3:** Relative changes in blood serum and urine parameters in rats following the exposure to selected element oxide nanoparticles compared to controls of the corresponding animal experiment, %.

Parameter	Element Oxide Nanoparticles						
	Al_2_O_3_	TiO_2_	SiO_2_	CdO	PbO	SeO	CuO
Serum Creatinine	↑0.6 (0.942)	↓11.59 (0.116)	↓7.00 (0.365)	↑6.60 (0.289)	↑6.79 (0.937)	*↓*15.44 * (0.017)	↓1.22 (0.774)
Serum Urea, mmol/L	↓16.89 (0.120)	↓12.39 (0.337)	*↓*25.9 * (0.044)	↑25.43 (0.283)	↑25.43 (0.504)	↓13.19 (0.566)	↓17.14 (0.399)
Urine Total Protein, mg/L	↑56.84 * (0.033)	↑13.72 (0.572)	↑33.71 (0.481)	↑10.69 (0.608)	↓12.58 (0.602)	↑62.54 (0.659)	↓29.76 * (0.009)
24 h Total Excretion of Protein, mg	↑11.68 (0.623)	↑6.93 (0.926)	↓1.82 (0.912)	↑14.29 (0.384)	↑18.59 (0.286)	↓43.85 * (0.017)	↓50.42 * (0.006)
Urine Creatinine, mmol/L	↑63.05 * (0.007)	↑36.94 (0.159)	↑59.24 (0.195)	↑12.43 (0.329)	↓1.69 (0.390)	↓16.46 (0.335)	↓20.73 (0.793)
Urine Protein Creatinine ratios, mg/μg	↓0.58 (0.988)	↓15.09 (0.358)	↓17.92 (0.332)	↑10.09 (0.534)	↑6.42 (0.609)	↓30.43 (0.098)	↓50.31 * (0.019)
Urine Urea, mmol/L	↑39.30 * (0.024)	↑21.26 (0.363)	↑37.23 (0.347)	↑11.70 (0.096)	↑12.10 (0.324)	↑29.35 (0.410)	↓67.89 (0.058)

Note: * Statistically different from the control groups (*p* in brackets, based on Student’s *t*-test), ↓—decrease, ↑—increase.

**Table 4:** Proportion of cells of different types in rat kidney imprint smears following exposure to element oxide nanoparticles, % ($\overset{-}{X}$ ± s.e.).

Exposure Group	Renal Proximal Tubular Epithelial Cells	Degenerated Cells of Proximal Tubules	Renal Distal Tubular Epithelial Cells	Degenerated Cells of Distal Tubules	Neutrophils	Eosinophils
Animal Experiment with Al_2_O_3_, TiO_2_, and SiO_2_ Nanoparticles						
Al_2_O_3_ NPs	60.33 ± 2.82	13.00 ± 1.94	7.00 ± 1.47	8.33 ± 1.60	4.33 ± 1.18	2.33 ± 0.87
TiO_2_ NPs	56.00 ± 2.87 *	14.00 ± 2.00	7.67 ± 1.54	8.33 ± 1.60	5.00 ± 1.26	5.00 ± 1.26 *
SiO_2_ NPs	56.67 ± 2.86 *	14.67 ± 2.04	9.00 ± 1.65	6.00 ± 1.37	5.67 ± 1.33	2.00 ± 0.81
Deionized Water (Control)	67.67 ± 2.70	10.00 ± 1.73	7.67 ± 1.54	5.00 ± 1.26	5.00 ± 1.26	0.67 ± 0.47
Animal Experiment with PbO and CdO Nanoparticles						
PbO NPs	69.20 ± 1.66 *	9.80 ± 0.66 *	6.83 ± 0.48 *	5.00 ± 0.58	5.00 ± 0.73 *	3.33 ± 0.95
CdO NPs	69.17 ± 1.38 *	8.33 ± 1.33 *	9.20 ± 0.37	4.67 ± 0.42	2.50 ± 0.43	1.50 ± 0.22
Deionized Water (Control)	75.00 ± 1.38	4.20 ± 0.37	9.00 ± 0.71	4.20 ± 0.37	2.40 ± 0.40	1.80 ± 0.58
Animal Experiment with SeO and CuO Nanoparticles						
SeO NPs	51.50 ± 1.45 *	17.33 ± 0.99 *	8.17 ± 0.60 *	6.83 ± 0.48 *	6.83 ± 0.48	4.33 ± 0.49 *
CuO NPs	55.67 ± 0.84 *	14.67 ± 0.49 *	8.33 ± 0.56*	6.67 ± 0.33 *	5.50 ± 0.43	4.33 ± 0.42 *
Deionized Water (Control)	64.33 ± 1.26	6.33 ± 0.49	11.33 ± 0.88	5.00 ± 0.58	5.50 ± 0.56	2.67 ± 0.33

Note: * Statistically different from the control group (*p* < 0.05, based on Student’s *t*-test).

## Data Availability

The data presented in this study are available on request from the corresponding author.

## Appendix A

**Table A1:** Some functional parameters of the excretory system of rats following 18 repeated intraperitoneal injections of suspensions of Al_2_O_3_, TiO, SiO, PbO, CdO, SeO, and CuO nanoparticles for six weeks ($\overset{-}{X}$ ± S.e.).

Index	Exposure Group									
	Deionized Water (Control)	Al_2_O_3_ NPs	TiO_2_ NPs	SiO_2_ NPs	Deionized Water (Control)	PbO NPs	CdO NPs	Deionized Water (Control)	SeO NPs	CuO NPs
Kidney Weight, g	1.99 ± 0.04	1.89 ± 0.04	2.01 ± 0.04	1.97 ± 0.02	1.85 ± 0.05	2.02 ± 0.06 *	1.88 ± 0.08	1.73 ± 0.04	1.85 ± 0.09	1.68 ± 0.06
Kidney Weight, g/100 g Body Weight	0.61 ± 0.01	0.56 ± 0.01 *	0.60 ± 0.01	0.60 ± 0.01	0.60 ± 0.01	0.66 ± 0.01 *	0.61 ± 0.02	0.44 ± 0.08	0.47 ± 0.08	0.43 ± 0.08
Uric Acid in Blood Serum, μmol/L	120.50 ± 10.86	154.88 ± 13.53	135.50 ± 15.29	128.25 ± 17.67	86.53 ± 6.91	87.43 ± 4.60	92.93 ± 8.08	132.50 ± 12.37	106.00 ± 13.42	153.43 ± 29.47
Serum Urea, mmol/L	4.44 ± 0.34	3.69 ± 0.28	3.89 ± 0.45	3.29 ± 0.40 *	4.64 ± 0.59	5.82 ± 0.68	5.82 ± 0.77	4.55 ± 0.75	3.95 ± 0.68	3.77 ± 0.52
Serum Creatinine, μmol/L	34.84 ± 2.09	35.05 ± 1.78	30.80 ± 0.71	32.40 ± 1.29	37.84 ± 2.35	40.34 ± 3.46	40.41 ± 2.52	39.18 ± 1.63	33.13 ± 1.35^*^	38.70 ± 0.63
Clearance of Endogenous Creatinine, mL/min	1.40 ± 0.15	1.58 ± 0.18	1.90 ± 0.24	1.74 ± 0.19	1.16 ± 0.08	1.12 ± 0.08	1.14 ± 0.11	1.32 ± 0.09	1.40 ± 0.12	1.53 ± 0.08
24 h Urine Volume, mL	29.67 ± 4.36	18.14 ± 3.63	26.43 ± 3.88	28.43 ± 5.73	26.47 ± 2.75	20.30 ± 3.02	25.50 ± 2.09	25.50 ± 4.63	14.29 ± 4.94	24.38 ± 4.99
Urine Protein, mg/L	190.43 ± 29.63	298.45 ± 32.35 *	216.55 ± 33.41	254.63 ± 76.97	222.41 ± 26.87	246.19 ± 31.59	194.43 ± 17.90	232.16 ± 16.08	377.35 ± 147.54	163.05 ± 12.33 *
24 h Total Excretion of Protein, mg	5.48 ± 1.14	6.12 ± 0.67	5.86 ± 0.66	5.38 ± 0.71	5.11 ± 0.65	5.84 ± 0.64	6.06 ± 0.64	7.16 ± 0.62	4.02 ± 1.03 *	3.55 ± 0.72 *
Urine Creatinine, μmol/L	1.57 ± 0.11	2.56 ± 0.27 *	2.15 ± 0.35	2.50 ± 0.61	1.77 ± 0.17	1.99 ± 0.19	1.74 ± 0.12	1.64 ± 0.15	1.37 ± 0.07	1.30 ± 0.06
Urine Protein Creatinine ratios, mg/μg	1.06 ± 0.14	1.06 ± 0.11	0.90 ± 0.04	0.87 ± 0.07	1.09 ± 0.07	1.20 ± 0.09	1.16 ± 0.11	1.61 ± 0.27	1.12 ± 0.18	0.80 ± 0.13 *
Urine Urea, mmol/L	229.30 ± 16.00	319.41 ± 29.85 *	278.06 ± 46.97	314.67 ± 78.85	227.37 ± 18.53	253.98 ± 18.82	254.89 ± 21.77	209.06 ± 25.24	270.41 ± 61.87	141.94 ± 14.81
Uric Acid in Urine, μmol/L	234.00 ± 38.97	319.50 ± 45.51	292.50 ± 105.70	304.71 ± 93.85	205.86 ± 21.46	208.58 ± 13.10	226.15 ± 17.06	173.20 ± 9.36	173.33 ± 44.33	160.33 ± 6.17

Note: * Statistically different from the control group (*p* < 0.05, based on Student’s *t*-test).

**Table A2:** Morphometric indices of damage to the epithelium of proximal convoluted tubules in rats following subchronic exposure to element oxide nanoparticles ($\overset{-}{X}$ ± S.e.).

Index	Exposure Group									
	Deionized Water (Control)	Al_2_O_3_ NPs	TiO_2_ NPs	SiO_2_ NPs	Deionized Water (Control)	PbO NPs	CdO NPs	Deionized Water (Control)	SeO NPs	CuO NPs
Brush Border Loss, %	1.49 ± 0.56	1.85 ± 0.47	3.61 ± 0.99 *	2.24 ± 0.58	6.70 ± 1.90	33.54 ± 5.44 *	45.18 ± 5.03 *	5.34 ± 0.65	17.39 ± 1.64 *	53.92 ± 5.41 *
Epithelial Desquamation, %	0.00± 0.00	0.15 ± 0.15	0.42 ± 0.36	0.30 ± 0.25	0.00 ± 0.00	21.73 ± 5.51 *	63.06 ± 12.09 *	0.00 ± 0.00	13.27 ± 4.75 *	58.42 ± 9.68 *

Note: * Statistically different from the control group (*p* < 0.05, based on Student’s *t*-test).
